# Associations between dietary intake, diet quality and depressive symptoms in youth: A systematic review of observational studies

**DOI:** 10.34172/hpp.2022.32

**Published:** 2022-12-10

**Authors:** Yiqi Wang, Jianghong Liu, Charlene Compher, Tanja V.E. Kral

**Affiliations:** ^1^University of Pennsylvania School of Nursing, Philadelphia, PA, USA; ^2^University of Pennsylvania Perelman School of Medicine, Philadelphia, PA, USA; ^3^Department of Family and Community Health, University of Pennsylvania School of Nursing, Philadelphia, PA, USA; ^4^Department of Biobehavioral Health Sciences, University of Pennsylvania School of Nursing, Philadelphia, PA, USA; ^5^Department of Psychiatry, University of Pennsylvania Perelman School of Medicine, Philadelphia, PA, USA

**Keywords:** Eating, Diet quality, Depression, Child Health, Adolescent health, Systematic review

## Abstract

**Background:** Depression is the third leading cause of worldwide disease burden among youth, and nutrition- and diet-related behaviors have been considered as an effective strategy for reducing the risk of depressive symptoms. This systematic review aims to examine associations between dietary intake and diet quality with depressive symptoms among youth.

**Methods:** In this systematic review, a search of scientific articles published between 2000 and 2021 was performed in four databases (CINAHL, Embase, PsycInfo, and PubMed) according to the PRISMA checklist. After applying inclusion and exclusion criteria, observational studies that focused on associations between micronutrient, macronutrient, food group intake, and diet quality and depressive symptoms among youth, ages 3 to 18, were selected for review.

**Results:** Thirty-two articles met the review criteria. Dietary intake of magnesium, vitamin B12, fiber, fruits, vegetables, and fish were consistently inversely related to depressive symptoms. However, the evidence of associations between intake of vitamins B6, C, D, and E, iron, copper, zinc, omega-3 fatty acids, carbohydrate, and dietary fat and depressive symptoms was mixed. Dietary effects on decreased depressive symptoms were more pronounced in children than adolescents. Additionally, most studies failed to adjust for potential confounding variables.

**Conclusion:** This review provides preliminary and comprehensive evidence for a relationship between dietary intake, diet quality, and depressive symptoms in youth. Although the results are heterogeneous and more research is needed, our findings indicate the importance of nutrition interventions for youth for decreasing depressive symptoms or for preventing further symptom exacerbation.

## Introduction

 In 2008, the World Health Organization (WHO) designated major depression as the third leading cause of the global burden of diseases and hypothesized that it will eventually become the first global disease burden by 2030.^1,2^ Approximately 10% to 20% of children and adolescents globally are diagnosed with mental health disorders, including depression.^3^ An epidemiological study found that 50% of mental health disorders occur by age 14 and 75% occur by age 24.^4,5^ Besides genetic influences, psychosocial risks such as family bereavement, separation and conflict, and maltreatment also increase the risk of depression among children and adolescents.^6^ Physiological changes in the brain, such as the hippocampus and amygdala growth, have been linked to an increased risk for the onset of depression in adolescence.^7^ Moreover, evidence has shown that both probiotics and prebiotics play an important role in mental health and mental disorders possibly by exerting an effect on the gut microbiota and influencing nervous system activity.^8,9^ Studies have also observed the early onset of major depressive disorder, which usually continues into adulthood, with adverse outcomes, such as poor academic performance and increased risk of substance abuse and suicide.^10^ Depression is clearly a major problem for youth in modern society. During childhood and adolescence, profound biological and physiological changes occur which can pose psychosocial risks that can lead to an early onset of depressive symptoms that persist into adulthood.

 Although the number of studies addressing this issue has increased substantially, recent systematic reviews have focused more on the adult population. Only two studies have systematically examined the association between dietary intake and/or diet quality and depressive symptoms in younger age groups.^5,11^ Existing reviews of youth have emphasized the relationship between an unhealthy diet and unhealthy eating habits, eating behavior, and depression/depressive symptoms. More attention has been paid to associations with overall dietary intake than to associations with intake or deficiencies of specific nutrients.^5,11^ Although depressive symptoms are common comorbidities of many chronic diseases,^12^ these two reviews^5,11^ excluded them as comorbidities, limiting evidence in this area. Further, researchers often used inconsistent search strategies, making it difficult to review the nutrition literature. For example, the terms “dietary pattern,” “diet quality,” and “dietary intake” are regularly used interchangeably. Even though diet quality is usually used to assess dietary diversity and compliance of dietary intake with dietary recommendations, definitions of diet quality vary, and composite and cut-off points of diet quality indices also vary.^13,14^ This systematic review identified and used all previously used interchangeable terms for dietary quality and intake-related terms and thoroughly categorized the evidence for a more comprehensive review. Given the gaps in the existing literature and the growing body of evidence in this area, this review aims to (1) examine associations between dietary intake, food group intake, and dietary quality with depressive symptoms among youth; (2) identify methodological limitations of existing studies; and (3) provide recommendations for future studies. Studies have also indicated a positive and direct relationship between nutrient intake and serum nutrient concentrations, particularly micronutrient intake.^15^ However, none of the existing systematic reviews included studies on physiological measures of nutrient status. Therefore, studies that assessed serum nutrient concentrations were included in this review besides self-reported dietary intake.

## Materials and Methods

###  Data sources and search strategy

 According to Cochrane’s Preferred Reporting Items for Systematic Reviews and Meta-Analysis (PRISMA) checklist,^16^ relevant articles were identified in four electronic databases: CINAHL, Embase, PsycInfo, and PubMed in May of 2021 (Supplementary file 1, Table S1). Studies published between January 2000 and May 2021 were included in this review. Search terms included “Nutritional Status OR Nutritional Sciences OR Nutritional Physiological Phenomena OR Nutrients OR Eating OR Dietary Intake OR Diet Quality OR Food Group Intake,” AND “Depression OR Depressive Disorder,” AND “Adolescent OR Child”.

###  Inclusion and exclusion criteria

 In the first step, YW reviewed the articles for eligibility based on the title and abstract, and then the full articles were reviewed based on the inclusion and exclusion criteria. After the search, studies were systematically screened by YW and verified by TVEK. Empirical studies published in English were eligible if they met the following criteria: a) used an observational design; b) examined associations between micronutrients, macronutrients, and/or food group intake and depressive symptoms or comorbid depression; c) assessed diet quality related to food and nutrient intake and its associations with depressive symptoms or comorbid depression; d) included participants from 3 to 18 years of age. The exclusion criteria were: a) investigated broad dietary patterns or diet types without assessing nutrient and/or food group intake; b) included participants > 18 years of age; c) were designed as a narrative review/systematic review/literature review/concept analysis/expert opinion; d) were randomized controlled trials (RCTs). RCTs were excluded because this review aims to identify, as a first step, the cross-sectional and longitudinal relationships between dietary intake and diet quality with depression-related symptoms, rather than evaluating the effects of dietary intervention on changes in depressive symptoms.

###  Study selection 

**Figure 1:**
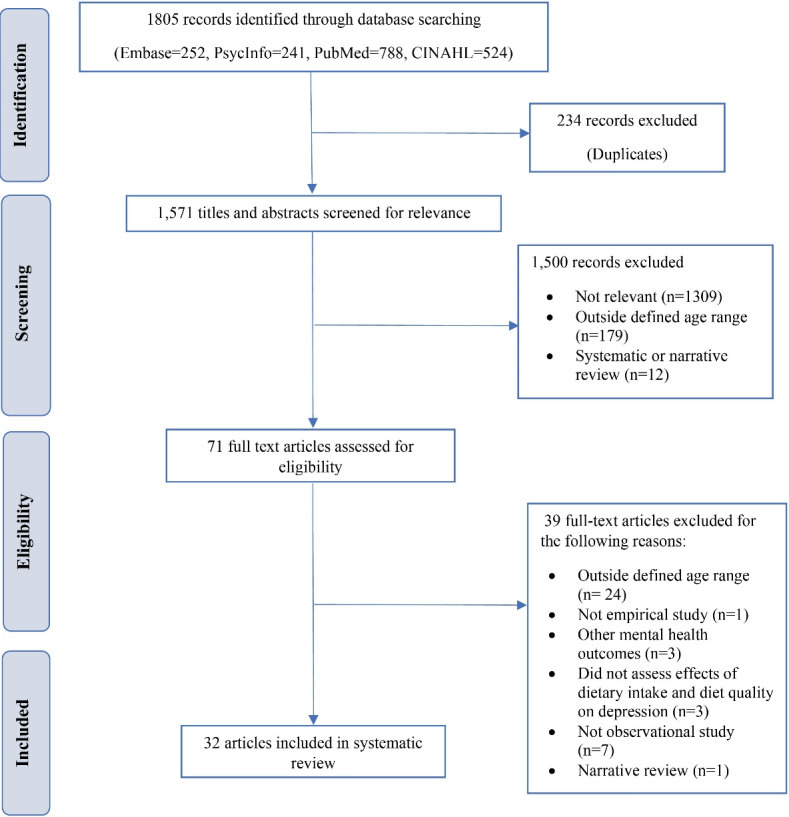
PRISMA flow diagram

 The PRISMA flow diagram (Figure 1) shows the article selection process.A total of 1805 articles were identified in the four databases. Of these, 1571 articles were screened for relevance by reviewing titles and abstracts after excluding 234 duplicate articles. An additional 1500 articles were further excluded, primarily because they did not address the relationship between dietary intake and depression. Of the 71 articles assessed for eligibility, 39 were excluded because they focused on participants outside our defined age range, included non-empirical studies, or did not examine the association between dietary intake and diet quality and depression. A total of 32 studies were included in this systematic review.

###  Data extraction and quality appraisal

 Data extracted from the articles included authors, years, countries, study designs (cross-sectional, longitudinal, cohort, and case-control studies), age group, sample size, gender, race/ethnicity, assessment instruments for dietary intake and depressive symptoms, and key findings. The methodological quality of each study (good, fair, or poor) was evaluated by YW using the National Institutes of Health (NIH) Quality Assessment Tool for Observational Cohort and Cross-Sectional Studies and Case-Control Studies.^17^

###  Concept definition

 The four key dietary variables in this review are daily micronutrient intake, macronutrient intake, food group intake, and diet quality (Table 1). Besides the dietary concepts, we focused on depressive symptoms rather than depression. Depressive symptoms refer to subthreshold depression, in which individuals exhibit core symptoms of depressed mood for at least two weeks without reaching the diagnostic threshold for clinical depression.^18^ Findings suggest that individuals with depressive symptoms are at an increased risk for subsequent mental health disorders and adverse health outcomes.^18^ Therefore, focusing on depressive symptoms rather than depression is a more inclusive and comprehensive approach that can help identify issues related to depression early and thus provide evidence for early prevention strategies.

**Table 1:** Definition of major concepts

**Key concepts**	**Definition**
Micronutrients	Micronutrients are referred to as vitamins and minerals.^19^
Macronutrients	Macronutrients include carbohydrates, protein and amino acids, fats and cholesterol, fiber, water, and energy.^20^
Food group	According to the MyPlate nutrition guide, the five food groups include fruits, vegetables, grains, protein, and dairy.^21^ Discretionary foods include sweet biscuits, cakes, desserts, and pastries; processed meats; sweetened condensed milk; ice cream and other ice confections; confectionary and chocolate; savory pastries and pies; commercial burgers and fried foods; potato chips, crisps, and other fatty and/or salty snack foods; cream, butter and spreads which are high in saturated fats; sugar-sweetened soft drinks and cordials, sports and energy drinks and alcoholic beverages.^22^
Diet quality	According to the United States Department of Agriculture (USDA), diet quality assesses how well a set of food intake conforms to the dietary recommendations based on the *Dietary Guidelines for Americans*, including diversity and variety in food choices.^13^

## Results

###  Study characteristics

 The table of evidence (Table 2) summarizes the study characteristics. The 32 studies included 22 cross-sectional studies, three case-control studies, and seven longitudinal studies (including two cohort studies).

**Table 2:** Table of evidence

**Author (year) ** **Country Study ** **design**	**Age, years [range; mean (SD)]**	**Sample size**	**Gender ** **(% female)**	**Race/Ethnicity**	**Depressive symptoms measurement tool**	**Dietary intake and diet quality measurement tool**	**Major findings (diet-depression relationship)**
Xu et al (2020)^1^ China Cross-sectional	10-20 14.9 (1.8)	n = 14 500	49.3%	NR	Self-report: CDI	Discretionary food intake: FFQ (self-report)	Consumption of fast foods and sugar-sweetened beverages was significantly associated with depressive symptoms in Chinese adolescents.
Gonoodi et al (2018)^15^ Iran Cross-sectional	12-18 15.2 (1.5)	n = 408	100%	NR	Self-report: BDI	Micronutrient intake: three-day food record (self-report)	Intake of dietary zinc was significantly higher in individuals without or with minimal depressive symptoms when compared to those with mild to severe depressive symptoms. Dietary zinc intake, but not serum zinc levels, was inversely associated with depressive symptoms.
Mrug et al (2021)^23^ USA Cross-sectional	11.1 at wave 1 13.1 at wave 2 16.1 at wave 3	Wave 1 n = 5147 Wave 2 n = 4773 Wave 3 n = 4521	51%	Black (34%) Hispanic (35%) Other minority (6%) White (24%)	Self-report: Major depressive disorder subscale from DPS	Food group and discretionary food intake: dietitian interview	No significant association between soft drink consumption and depressive symptoms among adolescents. More frequent intake of soft drinks at age 13 was associated with fewer depressive symptoms at age 16.
Liu et al (2020)^24^ 21 Low- and middle-income countries Cross-sectional (secondary data analysis)	12-15	n = 65 267	40.5% - 57.9%	NR	Self-report: GSHS	Food group intake: GSHS(self-report)	Inadequate intake of fruits and vegetables was significantly associated with a higher risk of depressive symptoms in Seychelles, Ecuador, Jordan, and Kenya, but only among males.
Khayyatzadeh et al (2020)^25^ Iran Cross-sectional	12-18 14.5 (1.52)	n = 988	100%	NR	Self-report: BDI	Micronutrient and macronutrient intake: FFQ(self-report)	Higher intakes of α-carotene, β-carotene, lutein, and vitamin C were significantly associated with a lower risk of depressive symptoms. Both soluble and insoluble dietary fiber intake was significantly higher in adolescents without depressive symptoms than those with depressive symptoms. No significant relationship between dietary intake of vitamin A and vitamin E and depressive symptoms.
Bahrami et al (2019)^26^ Iran Cross-sectional	12-18 14.5 (1.5)	n = 563	100%	NR	Self-report: BDI	Serum micronutrient levels	No significant differences between serum levels of vitamin A and E and depressive symptoms.
Khayyatzadeh et al (2019)^27^ Iran Cross-sectional	10-14 14.5 (1.5)	n = 750	100%	NR	Self-report: BDI-II	Food group intake: FFQ (self-report)	No significant associations between a traditional dietary pattern (high intake of potatoes, snacks, hydrogenated fats, vegetable oil, sugar, soft drinks, sweets, desserts, tea, salt, and spices) and a Western dietary pattern (high intake of refined grains, snacks, red meats, poultry, fish, organ meats, pizza, fruit juices, industrial juices and compotes, mayonnaise, nuts, soft drinks, sweets and desserts, coffee, and pickles) and depressive symptoms.
Tanaka and Hashimoto (2019)^28^ Japan Cross-sectional	Junior high school; 14 (0.86) Senior high school; 17.1 (0.88)	Junior high school n = 441 Senior high school n = 417	54.7%	NR	Self-report: CES-D	Food group intake: self-report questionnaire (breakfast and dietary intake)	Consumption of green and yellow vegetables once or more times per day was significantly inversely related to depressive symptoms among adolescents.
Ferrer-Cascales et al (2018)^29^ Spain Cross-sectional	12-17 14.3 (1.52)	n = 527	54.5%	NR	Self-report: CES-D	Diet quality: KIDMED (self-report)	Breakfast skippers had significantly lower levels of depression than those who ate a poor or very poor-quality breakfast (e.g., consuming commercially baked food). Adolescents who consumed a high-quality breakfast (e.g., bread, toast, cereal, and dairy products) had significantly lower levels of depression than those who consumed a poor-quality breakfast.
Khayyatzadeh et al (2018)^30^ Iran Cross-sectional	12-18 14.8 (1.5)	n = 535	100%	NR	Self-report: BDI	Micronutrient and food group intake: dietitian interview	High adherence to a DASH-style diet, including a higher intake of carbohydrates, dietary fiber, vitamin A, vitamin C, vitamin D, folate, calcium, magnesium, and potassium, was significantly associated with a lower risk of having depressive symptoms.
Neshatbini Tehrani et al (2018)^31^ Iran Cross-sectional	15-18 16.2 (0.97)	n = 263	100%	NR	Self-report: DASS-21	Food group intake: FFQ (self-report)	Higher adherence to the Mediterranean dietary pattern was significantly negatively associated with depressive symptoms.
Yu et al (2018)^32^ China Cross-sectional	9-11 9.8 (0.7)	n = 188	22.3%	NR	Self-report: DSRSC	Food group intake: self-administered questionnaire (derived from the Global School-Based Student Health Survey)	Adequate intake of fruits and vegetables was associated with a significant reduction in the risk of depressive symptoms.
Singh et al (2017)^33^ USA Cross-sectional	14-19 18 (1.2)	n = 114	100%	NR	Self-report: RADS	Micronutrient and macronutrient intake: ASA (self-report)	Higher intake of carbohydrates and fat was significantly associated with more depressive symptoms. A significant negative association was found between magnesium intake and depressive symptoms when controlling for total energy intake.
Sinclair et al (2016)^34^ Fiji Cross-sectional	13-18 15.6 (1.4) at baseline 17.4 (0.9) at follow-up	Baseline n = 7237 Follow- up n = 2948	Baseline 52.6% Follow- up 56%	Indigenous Fijian (42.5%) Indo-Fijian (52.4%) Other (5.1%)	Self-report: PedsQL^TM^ 4.0	Diet quality: ABAKQ	A higher diet quality, including fruits available at home, daily servings of fruits and vegetables, eating fruits after school, and consuming fruit drinks on school days, was significantly associated with lower scores of depressive symptoms at ages 15.6 and 17.4.
Rubio-López et al (2016)^35^ Spain Cross-sectional	6-9 8.21 (1.32)	n = 710	49.73%	NR	Self-report: CES-D	Micronutrient and macronutrient intake: 3-day food records (parent report)	Significant inverse relationships were found between higher dietary intakes of protein, carbohydrates, pantothenic acid, biotin, vitamin B12, vitamin E, zinc, manganese, cobalt, aluminum, thiamin, vitamin K, vitamin C, magnesium, iron, and bromine and depressive symptoms in children. A higher intake of fiber was significantly positively associated with depressive symptoms in children.
Richards and Smith (2015)^36^ UK Cross-sectional	11-17 13.6 (1.49)	n = 2307	51.5%	White (97.2%)	Self-report: Wellbeing Process Questionnaire	Food group and discretionary food intake: DABS (self-report)	A significant inverse relationship between caffeine intake and depressive symptoms in females but not in males.
Modan-Moses et al (2015)^37^ Israel Cross-sectional	14-18 16 (2)	n = 87	93.1%	NR	Clinical interview for DSM-IV: BDI	Micronutrient intake: 72-hour dietary recall (dietitian report) Serum micronutrient levels	No significant differences in vitamin D levels of patients with and without eating disorders and depressive symptoms.
Smith et al (2014)^38^ USA Cross-sectional	7-17 12.1 (3.1)	n = 38	52.6%	White (94.7%)	Self-report: CDI	Serum micronutrient levels	Lower serum vitamin D levels were significantly associated with increased depressive symptoms in youth with cystic fibrosis.
Weng et al (2012)^39^ China Cross-sectional	11-16 13.21 (0.99)	n = 5003	47.9%	NR	Self-report: DSRS	Food group and discretionary food intake: FFQ (self-report)	High intake of animal foods and snacks was significantly associated with depressive symptoms (without anxiety) among adolescents.
Jacka et al (2010)^40^ Australia Cross-sectional	10-14 11.6 (0.81)	n = 7114	57.3%	NR	Self-report: SMFQ	Diet quality: 14-item dietary questionnaire (dietitian graded)	A significant inverse relationship between a healthy diet score (i.e., intake of breakfast every day, low-fat dairy food intake, consumption of fruits and vegetables every day) and depressive symptoms was shown both before and after adjustment for confounding variables, such as age, gender, physical activity, and fathers' employment status.
Murakami et al (2010)^41^ Japan Cross-sectional	12-15 12 (6.5)	n = 3067	68.8%	NR	Self-report and parent report: CES-D	Micronutrient intake: BDHQ (self-report and parent report)	Higher intake of dietary B vitamins, especially folate and vitamin B6, was associated with a lower risk of depressive symptoms.
Murakami et al (2010)^42^ Japan Cross-sectional	12-15 Boys 12 + 6.5 Girls 13.5 + 8	n = 6517	52.9%	NR	Self-report: CES-D	Macronutrient and food group intake: BDHQ (self-report and parent report)	A higher intake of fish, EPA, and DHA was significantly and independently associated with a lower prevalence of depressive symptoms among males but not females.
Swann et al (2021)^43^ Australia Longitudinal	Baseline 14 Follow up 17	Baseline n = 1260 Follow- up n = 653	48% at 14 54% at 17	NR	Self-report: BDI-Y	Macronutrient intake: FFQ (self-report)	A high intake of dietary fiber was significantly inversely associated with depressive symptoms among adolescents.
Oddy et al (2018)^44^ Australia Longitudinal	Baseline 14 Follow up 17	Baseline n = 843 Baseline n = 838	51%	White (88%)	Self-report: BDI-Y	Food group and discretionary food intake: FFQ (parent report)	A high intake of a Western-style diet, which included red meat, take-out foods, refined foods, and confectionary foods at age 14 was significantly inversely associated with depressive symptoms at age 17.
Winpenny et al (2018)^45^ UK Longitudinal (Cohort study)	Baseline 14.5 (3.5 months) Follow up 17.5 (4.1 months)	Baseline n = 1238 Follow- up n = 932	60%	NR	Self-report: MFQ	Diet quality and food group intake: MDS and four-day diet diary (self-report)	No significant relationship between diet quality, intake of fruits and vegetables, intake of fish at age 14 years, and depressive symptoms at age 17 years. No significant cross-sectional association between diet quality or intake of fish and depressive symptoms.
Black et al (2015)^46^ Australia Longitudinal	Baseline 14 Follow up 17	Baseline n = 667 Follow-up n = 607	53.4%	NR	Self-report: YSR	Micronutrient intake: FFQ (self-report and parent report)	A significant inverse association was found between magnesium intake and depressive symptoms. No significant relationship between dietary intake of zinc and depressive symptoms.
Tolppanen et al (2012)^47^ USA Longitudinal (Cohort study)	Baseline 10.6 (NA) Follow up 13.8 (NA)	Baseline n = 2759 Follow-up n = 2752	NR	NR	Self-report: MFQ	Serum micronutrient levels	Higher serum levels of vitamin D3 were associated with a lower risk of depressive symptoms at age 13.8 but not at age 10.6. No significant association between vitamin D2 and depressive symptoms at either age 10.6 or 13.8.
McMartin et al (2012)^48^ Canada Longitudinal	10-11	n = 3757	52%	NR	Self-report: ICD-9 and ICD-10	Diet quality: FFQ (self-report) and DQI-I	Diet quality was not significantly associated with depressive symptoms. Greater dietary variety was associated with a significantly lower rate of receiving a diagnosis of depression. No significant association between dietary intake of vegetables, fruits, vitamin B6, folate, vitamin B12, and n-3 fatty acids and depressive symptoms.
Oddy et al (2011)^49^ Australia Longitudinal	Baseline 14 Follow up 17	Baseline n = 1376 Follow-up n = 977	50.9%	NR	Self-report: BDI-Y	Macronutrient intake: FFQ(parent report)	A significant negative association between dietary intake of EPA, VLC-PUFA, n-6 dihomogamma-linoleic acid (dGLA), AA, and adrenic acid and depressive symptoms at age 14. Significant inverse correlation between dietary intake of ALA, EPA, VLC-PUFA, total omega-3 polyunsaturated fatty acid (total n-3 PUFA), n-6 eicosadienoic acid, dGLA, AA, and adrenic acid and depressive symptoms at age 17. Dietary intake of total saturated and monounsaturated fat was significantly inversely associated with depressive symptoms at both ages 14 and 17.
Kim et al (2015)^50^ Korea Case-control	12-18 15 (1.5)	n = 116	100%	NR	Self-report: K-BDI	Micronutrient and food group intake: FFQ (self-report)	Higher consumption of fast foods including ramen noodles, hamburger, pizza, and fried foods and processed foods including ham, fish paste, and snacks were significantly associated with a higher risk of depressive symptoms. Dietary intakes of green vegetables and 1 to 3 servings of fruits per day were significantly associated with decreased risk of depression. Depressive symptoms were significantly negatively associated with intakes of fiber, b-carotene, vitamin B6, vitamin E, vitamin C, potassium, zinc, folate, iron, and copper.
Tsuchimine et al (2015)^51^ Japan Case-control	11-19 16 (2.2)	n = 24	100%	NR	Self-report: BDI-II, DSRSC	Serum micronutrient levels	A significant inverse relationship between serum levels of AA, DHA, and folate and depressive symptoms.
Benko et al (2011)^52^ Brazil Case-control	9-12 9.9 (1.0)	n = 51	11.8%	NR	Self-report: CDI	Food group and discretionary food intake: NBI (self-report)	Caffeine intake is significantly associated with a higher risk of having depressive symptoms after adjusting for sugar intake.

Abbreviation: NR, not reported; DPS, Diagnostic Interview Schedule for Children Predictive Scales; GSHS, Global School-based Health Survey; BDI, Beck Depression Inventory; FFQ, Food Frequency Questionnaires; CDI, Children’s Depression Inventory; BDI-II, Beck Depression Inventory, second edition; CES-D, Center for Epidemiologic Studies Depression scale; KIDMED, Mediterranean Diet Quality Index for children and teenagers; DASS-21, Depression, Anxiety, Stress Scale-2; DSRSC, Depression Self Rating Scale for Children; RADS, Reynolds Adolescent Depression Scales; ASA24, Automated Self-Administered 24-hour Dietary Recall; PedsQL^TM^, Pediatric Quality of life Inventory 4.0 Generic Core Scales; ABAKQ, Adolescent Behaviors, Attitudes and Knowledge Questionnaire; DABS, Diet and Behaviour Scale; DSM-IV, Diagnostic and Statistical Manual of Mental Disorders, fourth edition; DSRS, Depression Self Rating Scale; SMFQ, Short Mood and Feelings Questionnaire; BDHQ, Brief Self-administered Diet History Questionnaire; BDI-Y, Beck Depression Inventory, youth; MFQ, Mood and Feelings Questionnaire; MDS, Mediterranean Diet Score; YSR, Youth Self-Report; ICD-9, International Classification of Diseases, ninth edition; ICD-10, International Classification of Diseases, 10th edition; DQI-I, Diet Quality Index–International; K-BDI, Korean version of the Beck Depression Inventory; NBI, Nutrition-Behavior-Inventor; EPA, eicosapentaenoic acid; DHA, docosahexaenoic acid; AA, arachidonic acid; VLC-PUFA, very long-chain omega-3 fatty acids; ALA, alpha-lipoic acid; DHA, docosahexanoic acid.

 Four longitudinal studies used Western Australian Pregnancy Cohort (Raine) Study data.^43,44,46,49^ The four secondary data analyses from the Raine Study examined dietary intake (i.e., fiber, omega-3 fatty acids, magnesium, and Western-style dietary intake, including consumption of meat, refined foods, and take-out meals) and were therefore included in this review. More than half of the studies (n = 20) focused on adolescents (12-18 years), four studies focused on children ( < 12 years), and eight studies used samples of children and adolescents. Geographically, the regions in which these studies were conducted were widespread. They included Australia (n = 5), Brazil (n = 1), Canada (n = 1), China (n = 3), Fiji (n = 1), Iran (n = 6), Israel (n = 1), Japan (n = 4), Korea (n = 1), the United States (n = 4), Spain (n = 2), the United Kingdom (n = 2), and a mix of low-and middle-income countries (n = 1).

 Most studies used convenience sampling (n = 22), while ten studies carried out random sampling. Participants were recruited from schools, hospitals, medical centers, research institutes, and the community. Sample sizes ranged from 24 to 65 267 with a mean of 4114 participants. After removing outliers, defined as any sample sizes below the first quartile or above the third quartile (497 or 4584 participants, respectively), the average sample size was 1487 participants.

 Of the 32 studies, eight reported on micronutrient intake, two reported on macronutrient intake, 11 reported on food group intake, and four focused on diet quality. Additionally, studies that reported on micronutrient and macronutrient intake (n = 3), macronutrient and food group intake (n = 1), micronutrient and food group intake (n = 1), food group intake and diet quality (n = 1), and micronutrient and food group intake and diet quality (n = 1) were included. Instruments used to assess dietary intake or dietary quality included three-day food records (n = 3), a four-day diet diary (n = 1), the automated self-administered 24-hour dietary recall (ASA24) (n = 1), food frequency questionnaires (FFQ) (n = 12), 24-hour dietary recalls (n = 1), a brief self-administered dietary history questionnaire (BDHQ) (n = 2), the Mediterranean Diet Quality Index for children and teenagers (KIDMED) (n = 1), the Diet and Behavior Scale (DABS) (n = 1), a Mediterranean Diet Score (MDS) (n = 1), the Diet Quality Index–International (DQI-I)(n = 1), the Global School-based Health Survey (GSHS) (n = 1), the Adolescent Behaviors, Attitudes and Knowledge Questionnaire (ABAKQ) (n = 1), the Nutrition-Behavior-Inventory (NBI) (n = 1), the Amherst Health and Activity Study Adult Survey of Child Health Habits questionnaire (n = 1), a 72-hour dietary recall (n = 1), one self-administered questionnaire for dietary intake (n = 2 ) and clinical interviews (n = 2). Most of the questionnaires were self-reported (n = 20), while six of the questionnaires were based on parent-report. Besides using questionnaires, blood sampling of micronutrient levels was conducted in five studies.^26,37,38,47,51^

 Depressive symptoms were measured using the Beck Depression Inventory (BDI) (n = 10), the Center for Epidemiologic Studies Depression (CES-D) scale (n = 2), the Depression Self Rating Scale for Children (DSRSC) (n = 3), the Reynolds Adolescent Depression Scale (RADS) (n = 1), the Youth Self-Report (YSR)(n = 1), the Mood and Feelings Questionnaire (MFQ) (n = 2), the Children’s Depression Inventory (CDI) (n = 3), the Wellbeing Process questionnaire (n = 1), the GSHS (n = 1), the Adolescent Self-Report Pediatric Quality of Life Inventory 4.0 Generic Core Scales (n = 1), the International Classification of Diseases (ICD) (n = 1), the Short Mood and Feelings Questionnaire (SMFQ) (n = 1), a Persian version of the Depression, Anxiety, Stress Scale-21 (DASS-21) (n = 1), the Major Depressive Disorder subscale from the DISC Predictive Scales (n = 1), and a clinician interview (n = 1). Parents completed one depression instrument while youth completed the remaining tools.

Tables 3 and 4 summarize the findings between dietary intake (i.e., nutrient and food group intake) and micronutrient serum level and depressive symptoms.

**Table 3:** Relationship between self-reported dietary intake and depressive symptoms

**Nutrient/Food group**	**Positive association**	**Negative association**	**No association**
Micronutrient			
Zinc		^15,35,50^	^46^
Magnesium		^30,33,35,46^	
Potassium		^50^	^30^
Calcium			^30^
Iron		^35,50^	
Copper		^50^	
Sodium			^30^
Vitamin A		^50^	^25,30^
Vitamin E		^35,50^	^38^
Folate		^41,50^	^30,48^
Vitamin B6		^41,50^	^48^
Vitamin B12		^35^	^48^
Vitamin C		^25,35,50^	^30^
Vitamin D			^30,37^
Omega-3 fatty acids		^49^	^48^
Macronutrient			
Carbohydrate		^35^	^30^
Protein	^39,44^	^27,31,42,45^	^30,45^
Fat	^33^	^49^	
Fiber	^35^	^25,29,50^	^30^
Food group			
Fruits	^24^	^24,27,28,31,32,39,44,45^	^30,45,48^
Vegetables	^24^	^24,27,28,31,32,39,50^	^45,48^
Dairy		^27,29,31^	^27^
Discretionary foods	^1,39,50,52^		
Discretionary drinks	^1^	^36^	^30^
Nuts		^31,39^	^30^
Legume		^31^	^30^
Whole grain		^31,39^	^30^
Fish		^27,31,41,45^	^30,45^
Red meat	^39,44^	^31^	

**Table 4:** Relationship between serum-level micronutrients and depressive symptoms

**Nutrient/Food group**	**Positive Association**	**Negative Association**	**No Association**
Micronutrient			
Vitamin A			^26^
Vitamin E			^26^
Folate		^51^	
Vitamin D		^38^	
Vitamin D2			^47^
Vitamin D3		^47^	

###  Study quality 

**Figure 2:**
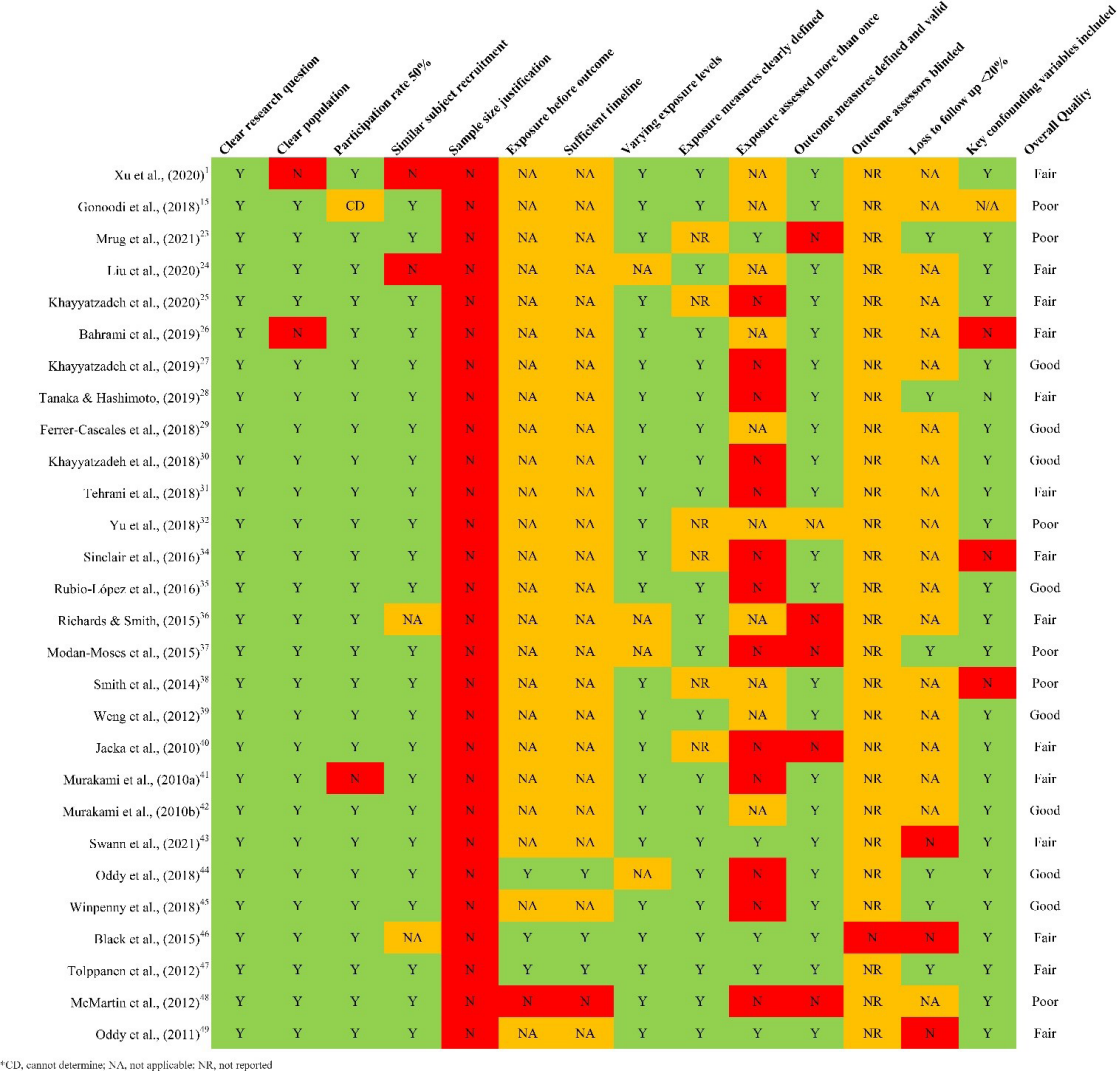
Quality appraisal for observational cohort and cross-sectional studies

**Figure 3:**
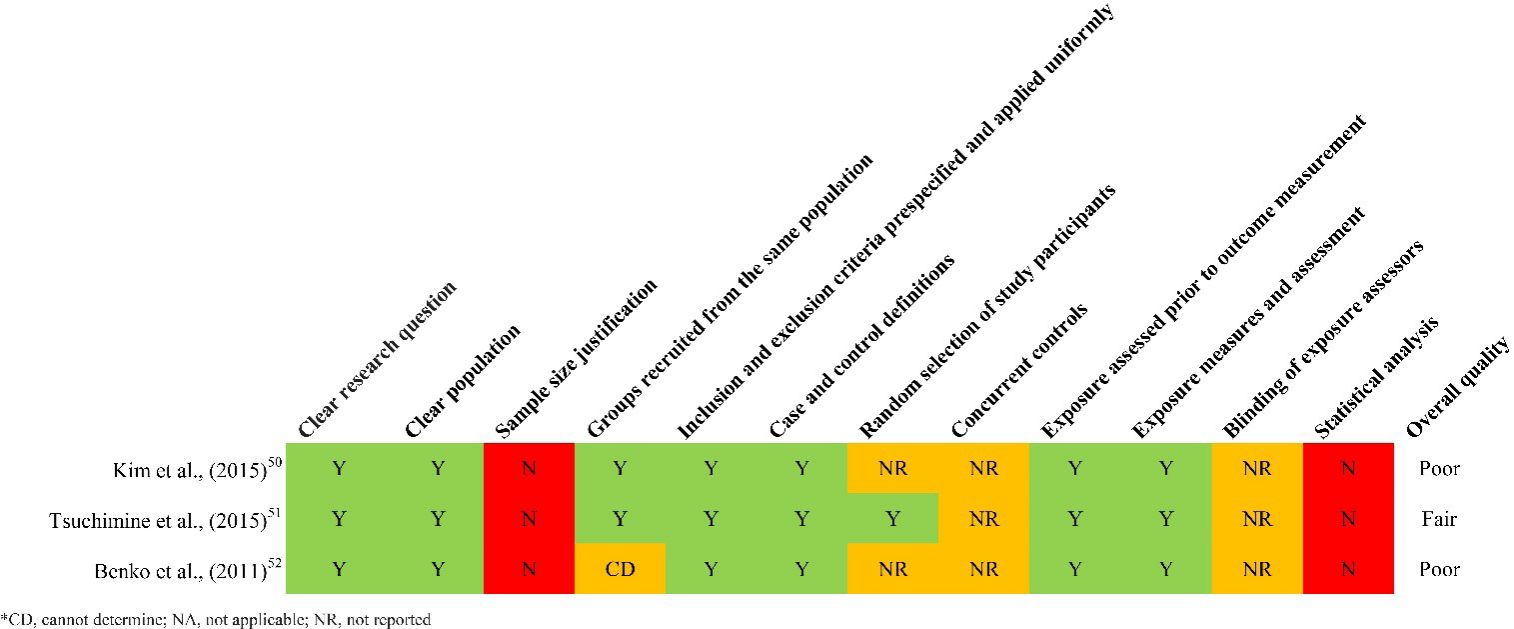
Quality appraisal for case-control study

 The overall quality of the selected articles was rated as fair (n = 16), good (n = 8), and poor (n = 8; Figures 2 and 3). Furthermore, ten studies failed to adjust for potential confounding variables, including socioeconomic status, behavioral factors (such as smoking, physical activity, and sleep), and family functioning, which can influence study outcomes. Additionally, participants’ race/ethnicity was reported in only five studies.

###  Relationship between micronutrient intake and depressive symptoms

####  Children 

 Two studies, including a cross-sectional and a cohort study, examined micronutrient intake or serum blood concentration and depressive symptoms in children.^35,47^ The cross-sectional study indicated a significant inverse relationship between vitamin B12, vitamin C, vitamin E, magnesium, and iron intake and depressive symptoms in children.^35^ Furthermore, a cohort study suggested that neither higher serum concentrations of 25-hydroxy-vitamin D2 [25(OH)D2] nor D3 [25(OH)D3] concentrations were significantly associated with decreased depressive symptoms in children.^47^

####  Adolescents 

 Nine studies, including cross-sectional studies (n = 7), a case-control study (n = 1), and a longitudinal study (n = 1), inconsistently reported significant inverse associations between dietary intake of zinc, magnesium, vitamin A, vitamin B6, folate, vitamin E, vitamin C, vitamin D, and omega 3-fatty acid, as well as serum levels of 25-hydroxy-vitamin D [25(OH)D], vitamin A, and vitamin E, and depressive symptoms in adolescents.^15,25,26,33,37,41,46,47,50^ Of the three studies that examined dietary zinc intake, one study reported no significant association,^46^ while other studies indicated a significant negative relationship between zinc intake and depressive symptoms.^15,50^ Additionally, a significant inverse relationship between dietary magnesium intake and depressive symptoms was found in three cross-sectional studies^30,33,35^ and one longitudinal study.^46^

 Dietary vitamin D intake was examined in only one study.^30^ When using a self-report FFQ, no significant association between dietary vitamin D intake and depressive symptoms was found.

 Three studies found that the relationship between dietary folate and vitamin B6 intake on depressive symptoms was inconsistent. Folate and vitamin B6 intake was independently associated with a lower risk of depressive symptoms in adolescents.^41^ The study among Korean female adolescents suggested a negative association between folate and vitamin B6.^50^ However, the one study found no association between folate intake and depressive symptoms.^30^

 A longitudinal study and one cross-sectional study found a consistent significant inverse association between omega-3 fatty acid intake, particularly eicosapentaenoic acid (EPA) and docosahexaenoic acid (DHA), and depressive symptoms.^42,49^ In the longitudinal study, the associations were found only in females ^49^ while the cross-sectional study^36^ indicated that EPA and DHA intake was only associated with depressive symptoms in male adolescents.

 Intake of other dietary micronutrients, including potassium, iron, and copper, was significantly negatively associated with depression risk in a Korean study^50^ while this association was not found in a study examining the Dietary Approaches to Stop Hypertension (DASH) diet.^30^

 A study of Korean female adolescents found an inverse relationship between dietary antioxidant intake (vitamins A, C, and E) and depressive symptoms.^50^ In the DASH diet study, no association was found between vitamins A and C intake and depressive symptoms.^30^ Additionally, when vitamin A and E serum levels were measured as all-trans-retinal and α-tocopherol, no association was found between these nutrients and depressive symptoms.^26^

 Two studies addressed the association between serum 25(OH)D concentrations and depressive symptoms.^37,47^ One study indicated no significant association between serum 25(OH)D levels (with an average value of 24.4 ng/ml, suggesting vitamin D insufficiency) was found in depressed individuals with or without eating disorders, such as anorexia nervosa or bulimia nervosa.^37^ Another study suggested that the level of 25(OH)D3 concentration was inversely associated with depressive symptoms.^47^

####  Children and adolescents combined

 Two studies included children and adolescents, and one study reported that youth with depressive symptoms had significantly lower serum folate concentrations than healthy controls.^51^ Another study reported an inverse association between serum 25(OH)D levels and depression in patients with cystic fibrosis.^38^

###  Relationship between macronutrient intake and depressive symptoms

####  Children 

 Only one article addressed the association between macronutrient intake and depressive symptoms in children.^35^ The study showed a significant inverse association between dietary protein and carbohydrate intake with depressive symptoms.^35^

####  Adolescents 

 Included in this evaluation were studies that examined carbohydrates, fat, protein, and fiber.^33,43,49^ One study showed that greater depressive symptoms were significantly associated with higher carbohydrate and fat intake.^33^ The study also found no significant association between protein intake and depressive symptoms. Furthermore, a cohort study indicated that saturated and monounsaturated, but not total polyunsaturated, fat intake was significantly negatively correlated with BDI-Y scores.^49^ A longitudinal report illustrated that a higher intake of all food sources of fiber, including cereal, fruit, and vegetable fiber, was significantly inversely related to depressive symptoms in males.^43^ However, this relationship became non-significant after adjustment for overall dietary intake, including intake of high-fiber foods, take-out and refined foods, red and processed meats, and whole-fat dairy products.

###  Relationship between food group intake and depressive symptoms 

####  Children

 Concerning depressive symptoms in children, food groups and discretionary food intake were assessed.^32,52^ A cross-sectional study reported that increased fruit and vegetable intake significantly reduced the risk of depressive symptoms in Chinese children with obesity.^32^

 A case-control study examined the association between consumption of sweets and caffeinated beverages and depressive symptoms and reported that only consumption of caffeinated beverages had a significant inverse relationship with depressive symptoms.^52^ Importantly, in this study, lifestyle variables, such as diet and physical activity, were not controlled for, as all subjects attended the same public schools and were lower-to-middle income.^52^

####  Adolescents

 One longitudinal study,^44^ one cohort study,^45^ and three cross-sectional studies^28,31,42^ examined the relationship between consumption of high-protein foods (e.g., fish and red meat) and depressive symptoms in adolescents. Findings related to associations between fish consumption and depressive symptoms were mixed. Two studies of female adolescents^30,31^ reported a significant inverse relationship between the two variables; however, another study indicated a correlation, but only in males.^41^ In contrast, a prospective cohort study found no correlations between fish consumption and depressive symptoms in 14-year-olds at the 3-year follow-up.^45^ A longitudinal study found a significant positive association between red meat consumption and depressive symptoms.^44^

 Results regarding the association between fruit and vegetable intake and depressive symptoms were inconsistent. Based on secondary data analysis of data from 21 low and middle-income countries, inadequate intake of fruits and vegetables was significantly associated with a higher risk of depressive symptoms, but only among male adolescents from four countries, namely Seychelles, Ecuador, Jordan, and Kenya.^24^ Additionally, a cross-sectional study indicated that consuming green and yellow vegetables one or more times per day significantly reduced the risk of depressive symptoms.^28^ Another cohort study exhibited a negative correlation between fruit and vegetable intake and depressive symptoms at age 14, but the study did not find a prospective association at age 17.^45^ Studies examining the Mediterranean dietary pattern and DASH-style dietary pattern indicated that higher intake of fruits, vegetables, whole grains, fish, nuts, legumes, and dairy products was associated with a lower risk of depressive symptoms.^30,31^ Moreover, a longitudinal study examining “healthy” dietary patterns involving a high consumption of fruits, vegetables, fish, and whole grains at age 14 was associated with a lower risk of depressive symptoms at age 17.^44^

 A cross-sectional and longitudinal study consistently found that high consumption of discretionary foods, including processed foods, refined foods, and sugar-sweetened beverages, was associated with an increased risk of depressive symptoms.^1,44^

####  Children and adolescents combined

 Studies conducted in both age groups examined associations with consumption of discretionary foods (such as fried and processed meat, chocolate, and candy), fruits, vegetables, fish, and dairy products. One study indicated that higher consumption of traditional Chinese cuisine (e.g., higher consumption of fruits, vegetables, and dairy products) was associated with fewer depressive symptoms.^39^ Correspondingly, a cross-sectional study among youth in Iran indicated that a healthy dietary pattern characterized by high consumption of legumes, vegetables, fruits, and dairy was significantly associated with fewer depressive symptoms.^27^

 Although some studies have reported a significant positive association between intake of discretionary foods and depressive symptoms,^1,36,39,50,52^ others, such as a cross-sectional study, found no association between soft drink (soda) consumption and depressive symptoms in youth aged 11, 13, or 16 years.^23^

###  Relationship between diet quality and depressive symptoms

####  Children 

 One study examined the relationship between diet quality and depressive symptoms in children.^48^ In this study, a higher diet quality score corresponded to greater diet variety, adequacy, moderation, and balance. The results showed that diet quality was not significantly associated with depressive symptoms. Notably, none of the food groups or nutrients, including consumption of fruits and vegetables, vitamin B6, vitamin B12, and omega-3 fatty acids, were significantly correlated with depressive symptoms.^48^

####  Adolescents 

 Two cross-sectional studies^29,34^ and one cohort study^45^ examined the relationship between diet quality and depressive symptoms. Two of the studies focused on fruit and vegetable intake.^34,45^ One study demonstrated that adolescents who consumed more fruits and vegetables (which corresponded to a healthy diet quality factor) compared to those who had a higher intake of discretionary foods (which equaled an unhealthy diet quality factor), had fewer depressive symptoms. The results were similar in both adjusted and unadjusted models.^34^ One study indicated that diet quality was based on intake of vegetables, legumes, fruits, nuts, whole grains, red and processed meat, fish, and the ratio of monounsaturated to saturated fat.^45^ The findings showed no significant associations between diet quality and depressive symptoms.^45^ The quality of breakfast was assessed in another cohort study, which measured diet quality through the KIDMED. In this study, a high-quality breakfast was characterized by the consumption of cereal and dairy products, which were significantly associated with fewer depressive symptoms.^29^

####  Children and adolescents combined

 One cross-sectional study assessed the association between diet quality and depressive symptoms in children and adolescents.^40^ The low diet quality score of this study was based on a higher intake of discretionary foods. The study reported an inverse relationship between a higher diet quality score and symptomatic depression before and after adjusting for covariates such as demographic and family factors.^40^

## Discussion

 This systematic review included 32 empirical studies on the relationship between dietary intake, diet quality, and depressive symptoms from childhood to adolescence. Notably, intakes of magnesium, B12, fruits, vegetables, and fish were more consistently inversely related to depressive symptoms from childhood through adolescence. At the same time, evidence for associations with vitamin B6, folate, vitamins C and E, iron, copper, zinc, omega-3 fatty acids, carbohydrates, and fat was inconsistent. The impact of nutrient and food group intakes on a lower risk for depressive symptoms was more pronounced in children than in adolescents. In addition, there was little evidence for a relationship between diet quality and depressive symptoms. However, a higher intake of fruits, vegetables, and fish tended to be associated with a lower risk of depressive symptoms, corresponding with the findings from the food group intake studies included in the review.

###  Dietary intake, diet quality, and depressive symptoms 

 Typically, micronutrients are required in small amounts in the body but play a significant role in tissue structure, enzyme metabolism, fluid balance, cellular function, and neurotransmission.^53^ Potential mechanisms underlying the antidepressant actions of zinc, magnesium, and selenium include elevated expression of hippocampal and cortical brain-derived neurotrophic factors, modulation of serotonin, dopamine, noradrenaline, circadian modulation, and inflammation attenuation.^54^ Besides minerals, vitamins may improve depressive symptoms. For example, deficiencies in vitamin B12 and folate can lead to elevated homocysteine levels, which have previously been linked to depression in adults.^55^ Although the underlying mechanisms between the effects of dietary micronutrient intake and depressive symptoms have been studied, the results are inconsistent.

 In contrast to the known association between macronutrient intake and brain health, results did not consistently indicate a significant relationship between macronutrient intake and depressive symptoms among youth after adjusting covariates. Neurotransmitters, such as serotonin, dopamine, noradrenaline, and adrenaline, are composed of proteins in the form of amino acids, and low dietary amino acid intake can negatively impact mood and mental health.^56^ Furthermore, extremely low dietary carbohydrate intake may be linked with depression because energy and glucose can modulate neurotransmitter levels. Thus, low carbohydrate consumption may exacerbate depressive symptoms or decrease wellbeing.^57,58^ The effects of macronutrient intake on depressive symptoms may also relate to the percentage of energy consumed from the various macronutrients. For example, when consuming the recommended percentage of energy from carbohydrates (i.e., between 45 and 60% of total energy intake), a 10% increase of calories consumed from protein was associated with a significant decrease in depressive symptoms.^59,60^

 Food group intake, including fruit, vegetable, dairy product, and protein (fish) intakes, was consistently aligned with the results of the micronutrient and macronutrient intake studies. The significant inverse association between fruit and vegetable intake and depressive symptoms among youth is also consistent with previous findings observed in systematic reviews among adults.^61^ Even though the effect of fruits and vegetables on depressive symptoms is not fully understood, it can partially be explained by the nutrient content in fruits and vegetables. For example, green leafy vegetables are a rich source of folate, antioxidant vitamins, minerals, and dietary fiber.^62^

 Evidence for diet quality and an association with depression is limited, and there was great variability across studies in how diet quality was assessed and defined. For example, one study only assessed breakfast,^29^ while others focused only on fruit and vegetable intake.^34,45^ The findings related to associations between diet quality and depressive symptoms were mixed for both childhood and adolescence, and it was challenging to interpret the evidence due to heterogenous study designs and the different methods used for measuring diet quality.

###  Adjustment of confounding variables

 Most selected articles were adjusted for covariates related to sociodemographic factors (such as gender and socioeconomic status), behavioral factors (such as smoking, physical activity, and sleep), psychosocial factors (such as quality of friendship and family functioning), and anthropometric measures (such as height and weight). One article did not control for covariates and assumed that all participants were from the same public schools and socioeconomic class.^52^ Although most studies controlled for multiple behavioral factors, sleep was only accounted for in two studies.^28,45^ Sleep plays an essential role in mood and wellbeing, and recent research has shown that insufficient sleep contributes to adverse psychological outcomes, including depressive symptoms.^63^ In addition, studies suggest that delayed sleep onset and reduced sleep quality may increase the risk of developing depressive symptoms in children and adolescents.^64,65^

 Adjusting for total energy intake is important in empirical nutrition studies because a person’s energy needs depend on their body size, metabolic rate, and physical activity level.^66^ Another important function of adjusting for total energy intake is to mitigate errors stemming from self-reported dietary intake.^66^ However, of the 32 articles, only ten controlled for energy intake as a covariate.^15,27,30,31,33,43,45,48-50^ Findings were inconsistent with and without adjustment for energy intake. For example, an inverse relationship between carbohydrate intake and depressive symptoms was no longer significant after adjusting for energy intake.^33^ Also, since the age of the study participants ranged from 14 to 19 years, food intake likely differed for older and younger youth due to their different body sizes and energy requirements.^67^ Therefore, adjusting for energy intake is essential.

###  Discussion of inconsistent results

 Besides the inconsistency in controlling for covariates across studies, differences in the age groups included across studies, assessment methods, and study designs may also account for inconsistent findings across studies. When comparing different age groups, we found that the potentially detrimental impact of inadequate intake of nutrients and food groups on the risk of depressive symptoms was more pronounced and consistent in children than in adolescents. The discrepancy may also be partly due to the different studies conducted in children and adolescents included in the review. Only four studies addressed micronutrients, macronutrients, or food group intakes in children, whereas 20 studies examined these intakes in adolescents. Eight studies assessed the association in both children and adolescents.

 The methodologies of assessing dietary intake varied across studies and included serum blood sampling, self-report and parent-report questionnaires, and dietitian-assisted interviews. These methodological differences may account in part for the inconsistent findings in this review. For example, a significant inverse association was found between dietary zinc intake and depressive symptoms when data were obtained from a 3-day self-report questionnaire. However, no association was found when data were assessed by serum zinc concentrations in the same study.^15^ A possible explanation for the conflicting result is that serum zinc concentrations are influenced by the time of day of blood collection and the subjects’ inflammatory status, medications, and hormone levels. Therefore, adjustment for covariates is critical when using this assessment method.^68^ The barriers to obtaining accurate serum blood collection results can also lead to inconsistent results. These include a selection of the correct trace element-free collection tube, available cold chain, appropriate sample transportation and storage, and the laboratory’s capacity to conduct the test.^69^ In addition, one study found no significant association between dietary zinc intake and depressive symptoms when dietary intake was assessed using a self-report FFQ^30^ in contrast to another study with conflicting findings.^50^ Data derived from predicting energy requirements in adults showed that the results were more accurate when using data from a dietitian interview rather than self-report. However, information on self-reported dietary intake is also collected during an interview with a dietitian.^70^ Since interviewing by a trained expert reduces the risk of errors in estimating portion sizes and omitting ingredients, the amount of over-reporting when using FFQs tends to be higher when the food intake is self-reported without assistance or interview with a registered dietitian.^70^ On the other hand, the data also show that respondent bias, including how respondents respond to avoid criticism or seek praise, can affect the level of accuracy of dietary intake data obtained via a dietitian-assisted interview.^71^ Also, studies indicate that parents tend to under-report their child’s depressive symptoms to a greater extent than children themselves.^72,73^ Although the reliability and validity of self-report MFQ and CDI have been confirmed in youth aged 9 to 17, other studies reported that the evidence is insufficient due to small sample sizes and a failure to exclude youth who had already been diagnosed with depression.^72-74^

 Concerning differences in study designs, most longitudinal studies (i.e., five out of seven studies) reported significant negative associations between dietary intake or diet quality and depressive symptoms in youth compared to cross-sectional studies (13 out of 22 studies). Notably, both study designs (cross-sectional and longitudinal) are observational in nature and have their unique advantages and disadvantages. Importantly, causal conclusions from observational data cannot be drawn from either design. Since longitudinal studies show changes over time and explore related events to outcomes of interest in the same individuals,^75^ longitudinal studies may be better positioned to identify the long-term effects of inadequate dietary intake or diet quality on depressive symptoms.

###  Recommendations for future studies 

 The amount of food consumed should be carefully examined in future studies because it is a major determinant of energy intake.^76^ Future studies also need to consider non-dietary factors, such as sleep, that increase the risk for developing depressive symptoms and the confounding effect of dietary intake on depressive symptoms stemming from these factors. Furthermore, major risk factors for depression among youth include the parental history of affective disorders, adverse childhood experiences, and sex/gender.^77^ Other important risk factors such as sibling relationships, socioeconomic status, race/ethnicity, life events, and other demographic factors, should also be considered in future studies.^77^ Although self-report questionnaires are convenient and can be used with large samples to assess dietary intake in epidemiologic studies, this assessment method also has drawbacks because it relies on subjective assessment of dietary intake, which can be prone to bias and reporting error. Future micronutrient intake studies should utilize more objective assessment techniques to better quantify the nutritional status of participants in observational studies. Additionally, it would be helpful to further explore the relationship between nutrient interactions and depressive symptoms in a future review. Based on the inconsistent assessment methods of diet quality, future studies should use validated methods to assess participants’ overall diet quality. Finally, additional longitudinal studies and RCTs should be conducted in the future to better understand the relationship between dietary intake, depression, and/or depression-related symptoms. Even in the presence of significant associations between dietary intake, diet quality, and depressive symptoms, causal inferences cannot be drawn. Although conducting an RCT in dietary-related studies is challenging, RCTs should be the goal for future investigations since it is the most robust design for determining cause-effect relationships between interventions, especially for micronutrient supplementation and health outcomes.^78^ Lastly, evidence indicated that using probiotics in folate bio-fortification of dairy products, such as yogurt, can alter chemically synthesized folic acid in individuals with folate deficiency.^79^ Therefore, future research should also focus on product development to increase natural micronutrient concentrations in healthy snacks consumed by youth.

###  Strengths and limitations

 Strengths of the current review include large sample sizes and the inclusion of males and females worldwide. This review provides an overview of the association between dietary intake, diet quality, and depressive symptoms among youth. It also provides a broader context for this important topic and outlines necessary steps for future studies, including optimized study design and assessment methods and the need to control possible confounders. The literature review has several limitations that should also be discussed. First, due to the inherent limitations of cross-sectional studies, causal inferences between dietary intake, diet quality, and depressive symptoms cannot be drawn in this review. Second, most studies relied on self-report questionnaires to measure dietary intake and depressive symptoms, which may affect the accuracy of the data and lead to inaccurate results. Moreover, the selected studies showed great heterogeneity in measuring aspects of dietary intake and quality, nutrient status, and depressive symptoms, further complicating the interpretation of the findings. Also, due to the pronounced heterogeneity of the measurements of the independent variables and outcome measures, for example, there are 30 different nutrients/food groups included in this review, we opted to conduct a systematic review and not a meta-analysis. Besides, the inclusion of depression as a comorbid condition and physical health problems (such as obesity and cystic fibrosis) may lead to further variability. For example, patients with obesity may have different metabolic parameters, leading to a range of risk factors for developing depressive symptoms, such as changes in appetite and altered eating behaviors^80^ or experiences of weight stigma.^81,82^ The overall quality of the selected literature was fair. A common limitation of most studies was that they failed to adjust for potential confounding variables.

## Conclusion

 Depressive symptoms are considered warning signs; therefore, it is crucial to recognize these signs and symptoms early to prevent further exacerbation. Thus, symptoms of depression in children and adolescents should not be ignored. The findings of this review have important implications for subsequent research, clinical practice, product development, and health policy. At research level, more research is needed which carefully performs a clinical evaluation of participants and controls for possible confounders to better clarify the role of nutrients and dietary intake in relation to depressive symptoms as well as better understand the mechanisms underlying this relationship. Future research should also more comprehensively examine the association between diet quality and depressive symptoms and study the role of naturally occurring nutrients in the diet (as opposed to dietary supplements) on mental health outcomes. At practical level, health practitioners should assess and monitor youth’s dietary intake or serum micronutrient concentrations, especially magnesium and B12, as part of a more comprehensive dietary assessment during their regular annual check-ups or during their medical visit. In addition, health practitioners can also tailor dietary advice and recommendations to youth’s nutrition status. Evidence also suggested that promoting treatment and reducing mental health incidence among youth by professionals alone is insufficient.^83^ Therefore, involving a multidisciplinary team of health researchers, health care professionals, schools and health care facilities, and the government may increase the effectiveness of preventing mental disorders.^83^ Additionally, educating parents about depression and a healthy diet is critically important.^84^ And lastly, government, school, and health care facilities should develop and improve nutrition education programs, such as developing a dietary guideline for individual with depressive symptoms, to promote a healthy diet and support mental health.

## Acknowledgements

 The authors acknowledge Richard James, a librarian at the University of Pennsylvania, for helping develop the search terms.

## Author Contributions

**Conceptualization:** Yiqi Wang, Jianghong Liu, Tanja V.E. Kral.

**Data curation: **Yiqi Wang.

**Formal Analysis: **Yiqi Wang.

**Investigation: **Yiqi Wang.

**Methodology:** Yiqi Wang.

**Project administration: **Yiqi Wang.

**Validation: **Tanja V.E. Kral.

**Writing – original draft: **Yiqi Wang.

**Writing – review & editing:** Yiqi Wang, Jianghong Liu, Charlene Compher, Tanja V.E. Kral.

## Funding

 There was no grant support related to this study.

## Ethical Approval

 As all data used in this systematic review have already been published, additional approval from the ethical committee was not needed.

## Competing Interests

 None.

## Disclaimer

 The authors claim that no part of this paper is copied from other sources.

## Supplementary Files


Supplementary file 1 contains Table S1.
