# Supplementary material for: Associations between dietary intake, diet quality and depressive symptoms in youth: A systematic review of observational studies
Source: Health Promot Perspect. 2022 Dec 10;12(3):249–65. doi: 10.34172/hpp.2022.32 (PMC9808911; doi:10.34172/hpp.2022.32)
Supplement: Supplementary file 1 — contains Table S1. [file hpp-12-249-s001.pdf]

Wang et al, **Health Promotion Perspectives,** 2022, 12(3), 249-265
doi: 10.34172/hpp.2022.32
https://hpp.tbzmed.ac.ir

Associations between dietary intake, diet quality and depressive symptoms in youth: A systematic review of observational studies

Yiqi Wang[1,2*], Jianghong Liu[3], Charlene Compher[4], Tanja V.E. Kral[4,5]

[1]University of Pennsylvania School of Nursing, Philadelphia, PA, USA
[2]University of Pennsylvania Perelman School of Medicine, Philadelphia, PA, USA
[3]Department of Family and Community Health, University of Pennsylvania School of Nursing, Philadelphia, PA, USA
[4]Department of Biobehavioral Health Sciences, University of Pennsylvania School of Nursing, Philadelphia, PA, USA
[5]Department of Psychiatry, University of Pennsylvania Perelman School of Medicine, Philadelphia, PA, USA

Supplementary file 1

Table S1. Search strings by database

Database	Search Strings
Embase	(('trace element'/exp OR micronutrient* OR ‘mineral intake’/exp) AND ('nutritional deficiency'/exp OR ‘vitamin deficiency’/exp OR deficien* OR insuffien*)) AND ('depression'/exp OR depressive) AND ([adolescent]/lim OR [child]/lim OR [infant]/lim OR [preschool]/lim OR [school]/lim OR [young adult]/lim OR adolescen* OR teenage*) AND [2000-2021]/py NOT 'conference abstract'/it
CINAHL	(MH "Micronutrients" OR micronutrient*) AND (MH "depression" OR depression OR depressive) AND (MH "Deficiency Diseases+" OR MH "nutritional deficiency" OR MH "Avitaminosis+" OR deficien* OR insufficien*)<br><br>AND (child or adolescent OR infant OR adolescence OR teenage*)
PsycINFO	MAINSUBJECT.EXACT.EXPLODE ("Nutrition") AND ("trace elements" OR micronutrient*) AND (MAINSUBJECT.EXACT.EXPLODE("Major Depression") OR depression OR depressive) AND (MAINSUBJECT.EXACT("Vitamin Deficiency Disorders") OR MAINSUBJECT.EXACT("Nutritional Deficiencies") OR deficien* OR insufficien*) AND (child or adolescent OR infant OR adolescence OR teenage*)
PubMed	("Micronutrients"[Mesh] OR "Micronutrients" [Pharmacological Action] OR "Trace Elements"[Mesh] OR "Trace Elements" [Pharmacological Action]) AND (depression OR "Depressive Disorder"[Mesh]) AND ("avitaminosis"[Mesh] OR "deficiency diseases"[Mesh] OR deficienc* OR deficient OR insufficen*) AND ("Adolescent"[Mesh] OR "Child"[Mesh] OR "Infant"[Mesh] OR "young adult" OR teenage* OR adolescen*) AND ("2000/01/01"[Pdat]:"2030/01/01"[Pdat])
